# How plant composition in margins influences the assemblage of pests and predators and its effect on biocontrol in melon fields

**DOI:** 10.1038/s41598-024-63985-x

**Published:** 2024-06-07

**Authors:** Juan Antonio Sanchez, Luis de Pedro, Elena López-Gallego, María Pérez-Marcos, María José Ramírez-Soria, Luis Gabriel Perera-Fernández, Joaquín F. Atenza

**Affiliations:** 1 Biological Control and Ecosystem Services Laboratory, Instituto Murciano de Investigación y Desarrollo Agrario y Medioambiental, C/Mayor S/N, 30150 La Alberca, Murcia Spain; 2 GIS and Remote Sensing, Instituto Murciano de Investigación y Desarrollo Agrario y Medioambiental, C/Mayor S/N, 30150 La Alberca, Murcia Spain

**Keywords:** Ecosystem services, Conservation biological control, Floral plantings, Hedgerows, Greening, Margin restoration, Yield, Ecology, Zoology

## Abstract

Many agricultural landscapes offer few resources for maintaining natural enemy populations and floral plantings have frequently been adopted to enhance biological pest control in crops. However, restored margins may harbour both pests and natural enemies. The aim was to compare the abundance of pests and natural enemies in three types of margins (unmanaged, sown herbaceous floral strips and shrubby hedgerows) as well as in adjacent melon fields. Besides, yield was compared among melon fields as way of testing the effect of the type of margin on biocontrol. The research was carried out during 2 years in twelve melon fields from four different locations in southern Spain. Arthropods were sampled periodically in margins and melon fields by visual inspections and Berlese extraction. Hedgerow and floral strips hosted higher numbers of both pests and predators than unmanaged margins. Besides, hedgerows had a similar or higher number of natural enemies than floral strips but lower number of pests. In just a few occasions, the type of margin had a significant effect on the abundance of pests and natural enemies in melon fields, but rarely there was consistency between the two growing seasons. No differences were found in yield. We hypothesised that the lack of association in the abundances of pests and natural enemies between margins and melon fields could be attributed to the overriding effects of the landscape and/or the internal population dynamics of arthropods in melon fields. Overall, shrubby hedgerows are more recommended than herbaceous floral strips.

## Introduction

Many agricultural crops are subjected to strong cyclic perturbations and provide temporal habitats for many forms of life and arthropods in particular^1^. In annual crops, most of the vegetation is eliminated after harvest and the environment loses the basic requirements for the maintenance of most species. In each growing season, the re-establishment of the communities of arthropods in crops depends on the immigration of individuals from other habitats and, therefore, it is greatly influenced by the characteristics of the surrounding environment^1–4^. This is the case for both, pests and their enemies, and the ideal situation from a pest management perspective would be that the environment harbours low pest populations and high numbers of natural enemies. In this respect, the early agricultural literature regarded natural habitats as a source of pests, while in recent decades the beneficial aspects of biodiversity have pervaded^5–9^. Natural habitats may indeed represent a greater source of pests than natural enemies and the spill over of predators and parasitoids from natural habitats to crops cannot be taken for granted^10–14^. The knowledge of how to manage biodiversity to enhance ecosystem services still lacks strong foundations and there are plenty of cases where increasing plant abundance and diversity around crops has failed to reduce pest populations^11,13,15–18^. However, numerous studies have shown that biodiversity decline in intensified agricultural landscape compromise pest control and, although no direct correlation between biodiversity and ecosystem services was found, pest control generally improved as biodiversity increased^15,16,19^.

The intensification of agriculture has transformed great expanses of natural areas into arable land and further reduced wild vegetation in the rural landscape by increasing field size to facilitate mechanization^20–22^. Additionally, there has been a decrease in plant species richness due to frequent tillage, fertilisation and herbicide use^21,23,24^. Habitat restoration through the management of farmland vegetation has been one of the main approaches adopted to enhance natural biological pest control^25,26^. For instance, agri-environmental schemes that include the conservation of biodiversity and landscape elements have been implemented in Europe to cope with environmental, ecological and socio-economic problems due to agriculture^27^. Building green infrastructures, such as floral strips and hedgerows, is one of the frequently adopted practices for the conservation of biodiversity, in particular that of natural enemies to enhance biocontrol and of pollinating insects for crop pollination^25,28–32^. Overall these practices have been reported to give positive results^15,16,25,33^. However, they have not always been as effective as expected, probably because restored hedges generally represent only a small fraction of the surface and therefore may have a low effect in comparison to the surrounding landscape, because they may host not only beneficial but also pests, and because the persistence of the populations of natural enemies in these infrastructures may ultimately depends on the surrounding environment^4,10,11,29,34^.

Floral plantings have been highly valued by the scientific community as a way to enhance biodiversity and conservation biological control in agricultural landscapes, but they have often been received with reluctance by growers and have not been generally adopted if not by obligation or in presence of subsidies^35,36^. Among other reasons, this is probably due to the fact that their economic impact on crops has rarely been assessed^36–38^. The contribution of natural biological control to agricultural production in terms of avoided pest damage is highly significant and unquestionable^39^. For instance, in melons, biological pest control has been presented as a viable strategy to replace chemical control in the south-east of Spain: natural enemies naturally colonising melon fields were shown to supress pest populations to similar or even lower densities than insecticides applications^40^. However, although the establishment of hedgerows in the areas where most of the open field melons are grown in south-eastern Spain is compulsory since 2019, the contribution of floral plantings to pest control remains unknown.

Melons are a temporal crop and the colonization of newly established fields by pests and natural enemies on each growing season depends on immigration from other habitats. Therefore, agroecological practices aiming to increase local plant diversity could have a strong influence on the population dynamics of both pests and their natural enemies in crop fields^16^. In the present research, in the first place, we were interested to determine the role of different margin types as hosts for pests and natural enemies. Three type of margins were assayed, including hedgerows of shrubby plants, sown herbaceous floral strips and unmanaged margins with spontaneous herbaceous vegetation (Supplementary Table S1). In the second place, we aimed to assess the effect of those margins on the abundance of pests and natural enemies in melon crops. Our working hypothesis was that managed margins (i.e., hedgerows and sown floral strips) would host a higher abundance of natural enemies than unmanaged margins, which would in turn increase the abundance of natural enemies and enhance biological pest control in melon fields. We also predicted differences in the community structure of arthropods between shrubby and herbaceous margins with a consequent effect on pest and natural enemy abundances in melon fields. The research was carried out in localities with different landscapes to account for this factor. Finally, to assess the pest control efficacy of the different management strategies, yield was compared among melon fields with the three types of margins. Yield assessment has been considered as the best way to test whether biocontrol differed among habitat restoration practices^9^.

## Material and methods

### Location and experimental setting

The present research was carried out during 2014 and 2015 in twelve experimental melon fields of 30 × 10 m each, situated in four localities with different landscape features in the Murcia province, south-eastern Spain: Dolores de Pacheco (Torreblanca, N37°46′37ʺ W00º53′50ʺ), Torre Pacheco (CIFEA, N37°44′25ʺ W00°58′00ʺ), Fuente Álamo (Arroyo, N37°44′22ʺ W00°58′04ʺ) and La Alberca de las Torres (IMIDA, N37°56′26ʺ W01°08′00ʺ). Dolores de Pacheco was an area of intensive agriculture with mainly vegetables grown both in open fields and protected crops (Supplementary Fig. S1A). Dolores de Pacheco was an area of intensive agriculture with mainly vegetables grown both in open fields and protected crops (Supplementary Fig. S1A). Torre Pacheco was an area of intensive agriculture with some edifications, abandoned fields, and remnants of forest and shrubby vegetation (Supplementary Fig. S1B). Fuente Álamo was a mix of intensive and extensive agriculture with abandoned fields and some areas with shrubby vegetation (Supplementary Fig. S1C). La Alberca de las Torres was an urban area intercalated with some cultivated land on the edge of a forest of *Pinus halepensis* Mill. (Pinaceae) with a rich understory of Mediterranean shrubs (Supplementary Fig. S1D). Three melon fields with an uncultivated margin of 20 × 5 m along their shorter side were established in each locality. The distance among fields within localities ranged from 300 to 800 m. Each field had five rows with 30 plants of green type melon (cv. Mural, Syngenta, Almería) each, with a separation of 2 m between lines and 1 m between plants within lines. In 2014, all fields were planted between March 24th and 31st, and they were harvested at the end of June. In 2015, the fields were planted between April 1st and 4th, and they were harvested in the first week of July. The Fuente Alamo melon fields were only planted in 2014. None of the fields were sprayed with insecticides, and only fungicides were applied when utterly necessary. The same calendar as for the biological control melon fields described at de Pedro et al.^40^ was applied. Weed control in melon fields was not necessary. Daily drip irrigation was used to water the melon plants.

Three types of green infrastructures were tested on the uncultivated field margins in each of the four localities: (1) unmanaged margins with spontaneous herbaceous plants (UM), (2) sown floral strips of herbaceous plants (SFS); (3) and hedgerows of shrubby plants (HGR) (Supplementary Table S1). No other planted margins were present in the landscape at least within a radius of 3000 m.

The managed floral strips were sown with a mixture of seeds from the Apiaceae, Asteraceae, Boraginaceae, Brassicaeae, Caryophyllaceae, Fabaceae, Lamiaceae and Ranunculaceae families (Supplementary Table S1). The seeds were sown manually by mid-October in 2013 and 2014. In the hedgerows, 1-year old seedlings (approx. 30 cm tall) from the families Lamiaceae, Fabaceae and Asteraceae were transplanted at the beginning of November in 2013 (Supplementary Table S1). The sown floral strips were irrigated once every 1 or 2 weeks, depending on the season. In the hedgerows spontaneous herbaceous plants were removed periodically while in sown floral strips they were left to grow (Supplementary Table S1). The plant species composition of the two managed margin was selected to provide continuous blossoming from spring to early summer, based on previous studies^41,42^.

### Sampling of the vegetation and arthropods in margins

The sampling of the vegetation and arthropods in margins was conducted fortnightly in 2014, between 12 May and 30 June, and weekly in 2015, between 28 April and 24 June. This work was done by four-five scouters in approximately one hour per margin. The percentage cover of blossoming and green cover was estimated for each plant species in each type of margin using a 2 × 2 m plastic-tube frame. The 2 × 2 m square was further divided in four 1 × 1 m^2^ squares with thin strings and each of these squares was virtually divided into four other squares at sampling. Three repeats were taken at random in each type of margin on each sampling date from each locality.

Arthropods were counted using hands-free magnifying glasses (1.8–4.8×) in an approx.10 cm green shoot, without flowers, from ten plant species in each type of margin on each sampling date from each locality. In plants with basal rosette of leaves (e.g., *Echium* spp., *Plantago* spp.), a leaf of similar size to the 10 cm shoots was sampled. The target phytophagous species were thrips, aphids potential melon pests (i.e., *Aphis* spp., *Myzus persicae* (Sulzer)—Hemiptera: Aphididae—and *Macrosiphum euphorbiae* (Thomas) —Hemiptera: Aphididae), whiteflies (i.e., *Bemisia tabaci* (Gennadius)—Hemiptera: Aleyrodidae), spider mites (*Tetranychus* spp.) (Supplementary Tables S2 and S3). As for natural enemies, the focus was on natural enemies that could interact with these pests, such as hemipterans, thrips, phytoseiids, cecidomyiids, coccinellids, chrysopids, parasitised aphids (mummies), spiders, ants, etc. (Supplementary Table S2 and S3). Cecydomiids were recorded only when they were in association with spider mites or aphid colonies. Whenever there were doubts about the identification of the arthropods, some specimens were collected and taken to the laboratory to be observed under a stereomicroscope. In addition, 10 inflorescences (approx. 10 cm long) were collected individually for each plant species and introduced in ziplock plastic bags and taken to the laboratory for the extraction of arthropods. The extraction was carried out using Berlese funnels for 24 h; the funnels contained a piece of cotton soaked in permethrin at the underside of the lid. The samples were collected in 10% ethanol and stored at 80% ethanol, next the specimens were counted and identified under a stereomicroscope.

The identification was carried out to the genus or species level, whenever it was possible. Ants, spiders, cecidomyiids and chrysopids were identified at the family level. The following references were used for the identification of arthropods: Iturrondobeitia^43^ (spider mites); Mound and Kibby^44^ and Lacasa and Llorens^45^ (thrips); Blackman and Eastop (aphids)^46^; Ouvrard and Martin^47^ (whiteflies); Péricart^48^ and Wagner^49,50^ (heteropterans); and Stary^51^ (parasitoids of aphids).

### Sampling of arthropods in melon fields

The melon fields were sampled to estimate the abundance of pests and natural enemies according to the protocol described by de Pedro et al.^40^ during spring and early summer of 2014 and 2015. The sampling of arthropods was carried out in the same dates as in margins. Two different sampling methods were used: (i) visual sampling of melon leaves and (ii) flower sampling through arthropod extraction using Berlese funnels. In the visual sampling (i), on each date, sixty plants were randomly selected in each melon field, where insects and mites were counted using hands-free magnifying glasses (1.8–4.8×) on the third or fourth leaf from the apex. Specimens were collected and taken to the laboratory to be observed under the stereomicroscope in case of doubt about their identification. Aphid mummies were also taken to the laboratory, and kept into Petri dishes in a climatic chamber at standard conditions until adult emergence to identify the species of parasitoids. In the sampling of flowers (ii), fifteen melon plants were randomly chosen in each field on each date, and one female flower per plant was picked. Flowers were introduced in ziplock plastic bags (one per field and date) and taken to the laboratory, where arthropod extraction was done using Berlese funnels for 24 h and processed as explained above for the sampling of plants in margins. In the second growing season, the sampling of flowers was carried out only during 3 weeks due to the lack of flowers. The identification of arthropods was carried out as explained in the previous section.

### Statistical analyses

The abundance of each taxon of arthropod in every type of margin in each date and locality was estimated using the following index:

$$AI=\sum_{i=1}^{n}{GC}_{i}{*Ns}_{i}+{BC}_{i}{*Nf}_{i}$$

where, *i* is plant species, *n* is the number of plant species, *GC*_*i*_ is the proportion of green cover for the *i* plant species, *Ns*_*i*_ is the average number of individuals per green shoot in the *i* plant species, *BC*_*i*_ is the proportion of blossoming of the *i* plant species, *Nf*_*i*_ is the average number of individuals per inflorescence in the *i* plant species (Supplementary Table S1). Therefore, the index was created to weigh the abundance of pests and natural enemies according to the representation of each plant species in margins, as they were composed of many plant species with different abundances.

Generalised linear mixed models (GLMM) were used to test for the effect of margin type (i.e., hedgerows, sown floral strips and unmanaged margins) and year, plus their interaction, as fixed factors, on the abundance index (*AI*) of the potential melon pests (i.e., aphids, phytophagous thrips, spider mites and whiteflies) and their main natural enemies (i.e., *Orius* spp., cecidomyiids, phytoseiids and predatory thrips) in margins. GLMM were run using the “lmer” function (“lme4 package”)^52^, with locality and week of sampling as nested random factors. The data were transformed by the square root to account for the deviation from normality. The χ^2^ and p-values for the fixed factors were obtained by the Wald test using the “Anova” function in the R “car” package^53^. The post hoc pairwise multiple comparisons was run using the estimated marginal means (EMMs) with the function “glht” in the “multcomp” package in R^54^.

GLMM were also run to test for the effect of margin type and year on the abundance of the same groups of arthropods on melon leaves using the “glmmPQL” function (“lme4 package”). In this case, locality, plant and week were used as nested random factors, and the negative binomial was used for explaining the distribution of errors. The χ^2^ and p-values in the Wald test, and post hoc pairwise multiple comparison were run as previously explained. The abundance of *Orius* spp., phytophagous thrips and predatory thrips (i.e., *Aeolothrips* spp.) on flowers was also tested as a function of margin type and year using the “glmmPQL” function. In this case, the abundance was expressed as number of insects per flower, locality and week were introduced as random factors in the models, and the log-normal distribution was used for explaining the distribution of errors. The effect of margin type and year on yield (kg per plant) was also assessed using the same functions and procedure with locality as random factor and the log-normal distribution.

The segregation of the three margins was assessed by Kruskal’s non-metric multidimensional scaling (NMDS) using the Bray–Curtis distance. The function "metaMDS" in the "vegan" package was used to perform NMDS on the annual average of the abundance index (*AI*) of the different phytophagous and predator species for each type of margin in each locality^53^. Thereafter, Permutational Multivariate Analysis of Variance (PERMANOVA) was carried out to test for the effect of localities and type of margin, as well as their interaction. PERMANOVA was run using the function “adonis” (“vegan” package) and the pairwise comparison among localities and field margins was performed using the “pairwiseAdonis” function in the “pairwiseAdonis” package^55^. NMDS was also used to determine the segregation of the melon fields with different type of margins as a function of the annual average abundance of phytophagous and predator species in melon fields. For the species with flower preference, such as Thysanoptera (with the exception of *Scolothrips longicornis* Priesner) and Anthocoridae (Hemiptera), the annual average densities per flower were used as dependent variables for the NMDS analyses. For the rest of the species (e.g., aphids, whitefly, spider mites, phytoseiids, cecidomyiids), the annual average per leaf was used instead.

### Ethical approval

This article does not contain any studies with human participants or animals performed by any of the authors. The seeds used for the sowing of the floral strips and seedlings used for the establishment of the hedgerows were provided by commercial licenced nurseries that comply with the institutional, national, and international guidelines and legislation.

## Results

### Abundance of pests and natural enemies in margins

**Figure 1:**
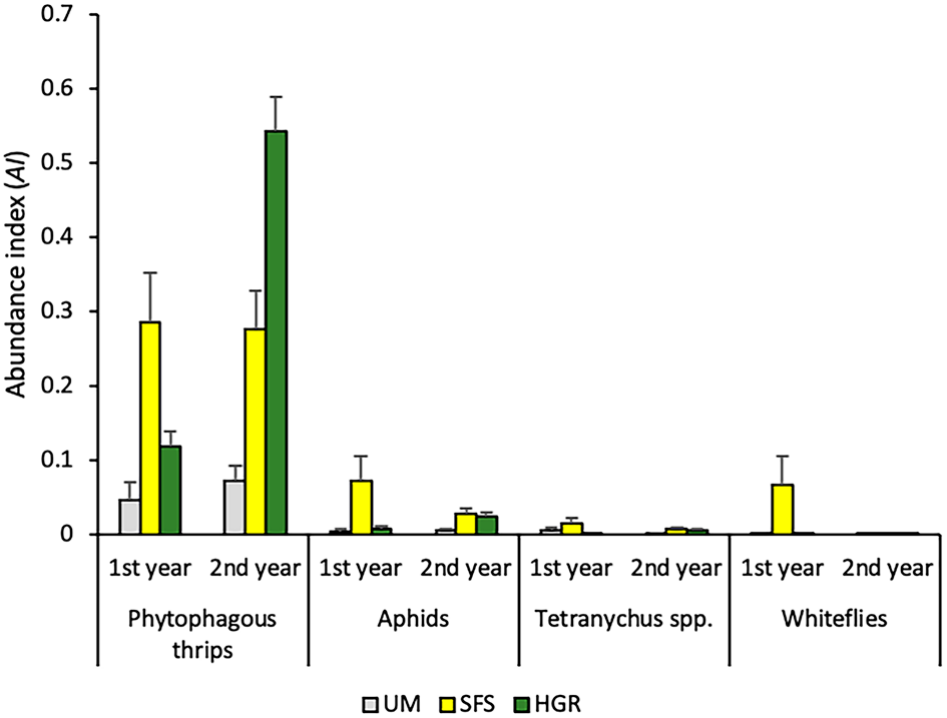
Average annual abundance index of pests in unmanaged (UM), sown floral strips (SFS) and shrubby hedgerows (HGR).

Phytophagous thrips were the most abundant pests in margins, followed by aphids, *Tetranychus* spp. and whiteflies (Fig. 1). The absolute abundances for the main pest taxa in margins are summarised in the Supplementary Table S2. Type of margin and year had a significant effect on the abundance of thrips, the interaction between the two factors also being significant (Table 1). The abundance index for the adults of *Frankliniella occidentalis* (Pergande) (Thysanoptera: Thripidae) accounted for 87.3%, 76.6% and 55.7% of the total number of phytophagous thrips found in sown floral strips (SFS), unmanaged margins (UM) and hedgerows (HGR), respectively. *Melanthrips fuscus* (Sulzer) (Thysanoptera: Aeolothripidae) (7.2%), *Thrips tabaci* Lindeman (Thysanoptera: Thripidae) (9.4%), *Thrips angusticeps* Uzel (Thysanoptera: Thripidae) (9.4%) and *Taeniothrips inconsequens* (Uzel) (Thysanoptera: Thripidae) (6.0%) were other less abundant thrips species found in margins. The type of margin had also a significant effect on the abundance of aphid pests, *Tetranychus* spp. and whiteflies (i.e., *B. tabaci*), being generally significant the interaction with year (Table 1). The abundance of pests and other phytophagous arthropods was generally significantly more abundant on managed (i.e., hedgerows and sown floral strips) than on unmanaged margins (Table 2).

**Table 1:** Statistics of Generalised Linear Mixed Models (GLMM) for the effect of margin type, year, and their interaction on the index of abundance of the main phytophages and predators in margins.

Group	Species	Margin type		Year		Interaction	
		χ^2^(2)	P-val	χ^2^(1)	P-val	χ^2^(2)	P-val
Phytophages	Thrips	101.7	< 0.001	1.83	0.176	50.9	< 0.001
	Aphids	21.2	< 0.001	2.28	0.131	9.44	< 0.01
	Mites	20.6	0.001	0.095	0.758	7.64	0.022
	Whitefly	9.40	< 0.01	3.68	0.055	5.58	0.061
Predators	Orius	68.0	< 0.001	30.4	< 0.001	18.0	< 0.001
	Thrips	83.7	< 0.001	8.70	< 0.001	39.6	< 0.001
	Cecidomyiids	36.9	< 0.001	8.15	< 0.01	25.8	< 0.001
	Phytoseiids	7.18	0.007	0.17	0.682	10.3	< 0.01

χ^2^ = Chi square (degrees of freedom within brackets).

**Table 2:** Summary of pairwise comparison for the abundance index of phytophagous and predator species among the three types of margins (shrubby hedgerows—HGR, sown floral strips—SFS, and unmanaged margins—UM).

Group	Taxa	HGD vs UM		HGD vs SFS		SFS vs UM	
		1st	2nd	1st	2nd	1st	2nd
Phytophagous	Mites	ns	ns	H < S***	ns	S > U**	S > Uº
	Thrips	H > U**	H > U***	H < S**	H > S***	S > U***	S > U***
	Aphids	ns	H < U*	H < S***	ns	S > U***	ns
	Whitefly	ns	ns	H < S**	ns	S > U**	ns
Predators	*Orius* spp.	H > U***	H > U***	ns	H > S***	S > U***	S > Uº
	Thrips	H > U*	H > U***	ns	H > S***	S > U*	S > U***
	Phytoseiids	ns	ns	ns	H < S***	ns	S > U**
	Cecidomyiids	ns	ns	H < S***	ns	S > U***	ns

(ns, not significant) P ≥ 0.1; (º) P < 0.1; (*) P < 0.05, (**) P < 0.05, (***) P < 0.01.

**Figure 2:**
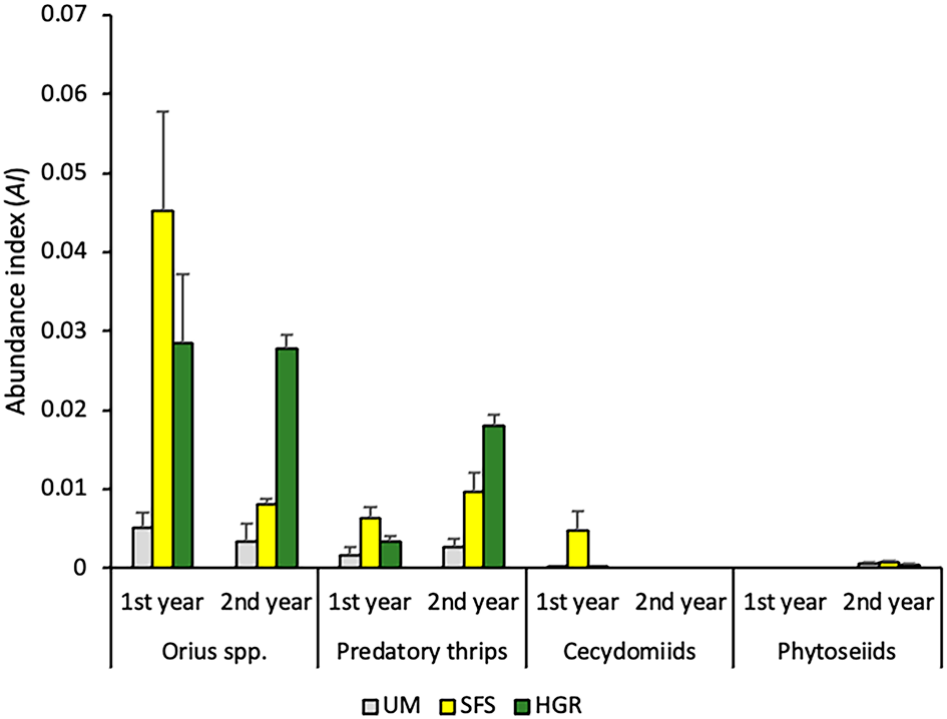
Average annual abundance index of predators in unmanaged (UM), sown floral strips (SFS) and shrubby hedgerows (HGR).

In relation to natural enemies, *Orius* spp. were the predators with the highest abundance in margins, followed by predatory thrips (i.e., *Aeolothrips* spp. and *S. longicornis*), cecidomyiids, and phytoseiids (Fig. 2). The absolute abundances for the main natural enemies in margins are summarised in the Supplementary Table S2. Type of margin had a significant effect on the abundance of all the groups of natural enemies, the effect of year and the interaction between the two factors being generally significant (Table 1). Predators were usually significantly more abundant on managed than on unmanaged margins (Table 2).

### Abundance and population dynamics of melon pests and their natural enemies in melon fields

#### Abundance and population dynamics of melon pests in melon fields

**Figure 3:**
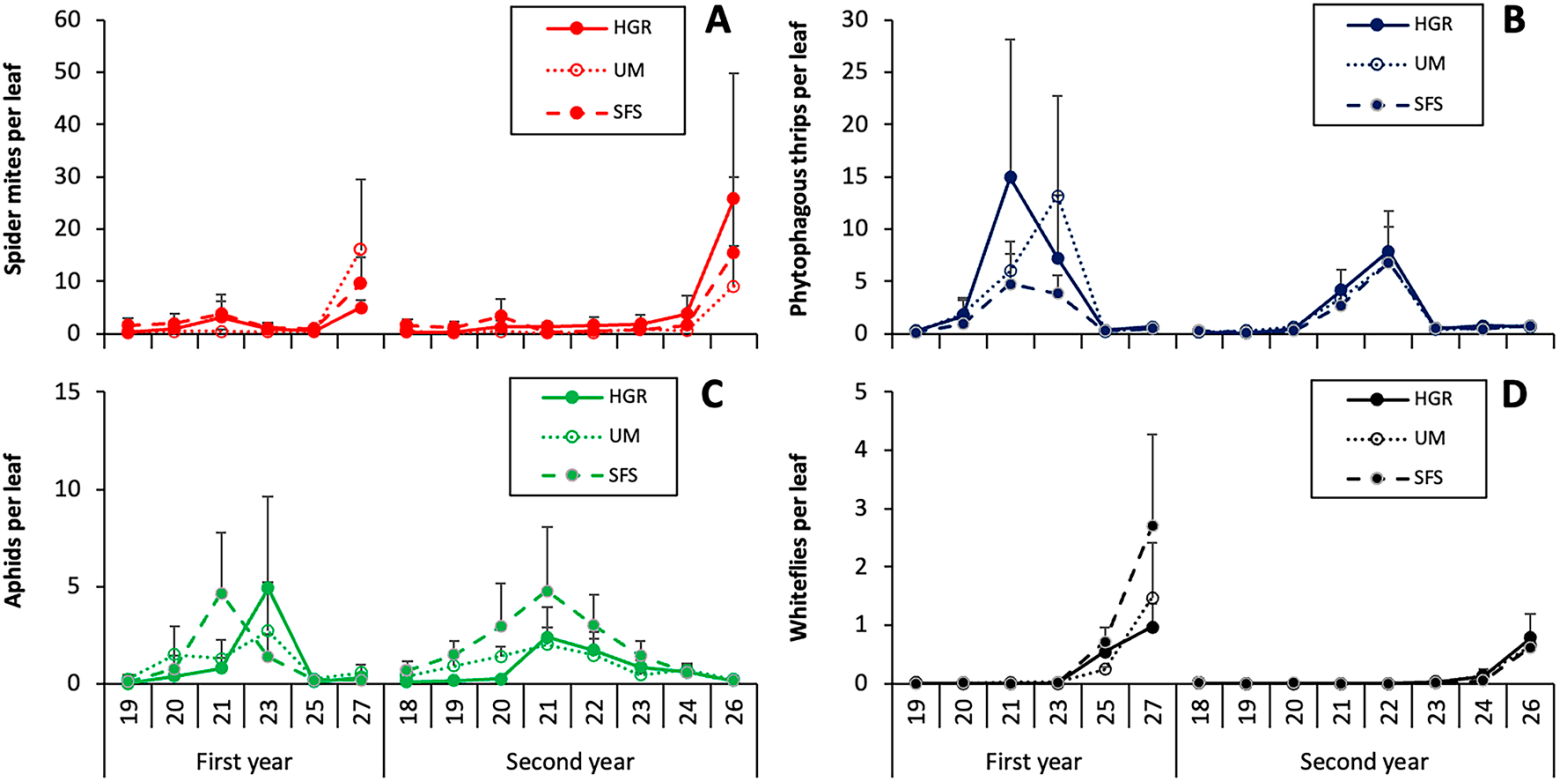
Abundance of the main pests observed in the leaves of melon plant in fields with unmanaged margins (UM), sown floral strips (SFS) and hedgerows (HGR), expressed as the average number of individuals per leaf ± SE: spider mites (**A**), phytophagous thrips (**B**), aphids (**C**) and whiteflies (**D**). Numbers above year’s labels indicate weeks.

In melon leaves, the most abundant phytophagous arthropods were spider mites (35.8% of the pests counted on leaves), phytophagous thrips (33.7%), aphids (27.6%) and whiteflies (2.9%). The absolute abundances for the main pests in melon fields are summarised in the Supplementary Table S3. The abundance of spider mites in melon fields was significantly influenced by the type or margin (χ^2^ = 31.3; df = 2; P < 0.001), with significant differences between the two years (χ^2^ = 11.8; df = 1; P < 0.001) and a significant interaction between type of margin and year (χ^2^ = 12.8; df = 2; P = 0.002). Spider mites showed a low abundance along most of the sampling period, but their abundance increased in the last weeks, both in the first and second year (Fig. 3A).

On the sampling of leaves, phytophagous thrips were mainly represented by *F. occidentalis* (90% of the thrips adults), with a low presence of other species such as *T. tabaci* and *M. fuscus*. Most of the thrips observed in the visual sampling were larvae (80%). The abundance of phytophagous thrips was significantly influenced by both margin (χ^2^ = 18.7; df = 2; P < 0.001) and year (χ^2^ = 24.2; df = 1; P < 0.001), with a significant interaction between the two factors (χ^2^ = 14.9; df = 2; P < 0.001). The abundance of thrips generally peaked between weeks 21st and 23rd (Fig. 3B). In the sampling of flowers, the phytophagous thrips species observed were *F. occidentalis* (93.5% of the adults), *T. tabaci* (2.9%) and *T. inconsequens* (3.6%). Thrips larvae represented 25.1% of the individuals in flowers. The abundance of phytophagous thrips in flowers was not significantly influenced by margin (χ^2^ = 2.72; df = 2; P = 0.257), but differed significantly between years (χ^2^ = 79.1; df = 1; P < 0.001). In the first year, phytophagous thrips reached similar density peaks on week 21st in the melon fields with the three types of margins (SFS: 14.7 ± 2.9; UM: 13.8 ± 6.5; HGR: 14.7 ± 4.1 individuals per flower ± SE). In the second year, the density peaks were below 7 individuals per flower in all the fields.

*Aphis gossypii* Glover (Hemiptera: Aphididae) represented 99.3% of the aphid species observed in the sampling of melon leaves. *Myzus persicae* (Sulzer) (Hemiptera: Aphididae) was found occasionally. The density of aphids in melon fields was significantly influenced by margin type (χ^2^ = 31.8; df = 2; P < 0.001) and year (χ^2^ = 56.0; df = 1; P < 0.001), the interaction being also significant (χ^2^ = 16.7; df = 2; P < 0.001). Aphids reached their maximum density between week 21st and 23rd (Fig. 3C).

*Bemisia tabaci* was the only whitefly observed in the melon fields. Most of the whitefly counted on melon leaves were nymphs (90.5%). The density of whiteflies significantly differed between years (χ^2^ = 25.3; df = 2; P < 0.001), but no significant differences were found among melon fields with different margins (χ^2^ = 2.23; df = 2; P = 0.329). In both years, the density of whitefly reached a maximum in the last week (Fig. 3D).

No significant differences were found on the pairwise comparison among the abundances of pests in melon fields with different margin types in most of the cases and rarely the results were consistent between the first and the second year (Table 3). Melon fields with hedgerows had a higher abundance of spider mites than those with unmanaged margins both in the first and the second year.

**Table 3:** Summary of pairwise comparison of the abundance of pests and predator species among melon fields with different types of margins.

Group	Sampling	Taxa	HGD vs UM		HGD vs SFS		SFS vs UM	
			1st	2nd	1st	2nd	1st	2nd
Pests	Leaves	Mites	H > U*	H > U*	H < S**	ns	S > U***	ns
	Leaves	Thrips	ns	ns	H > S***	ns	S < U***	ns
	Flowers	Thrips	ns	ns	ns	ns	ns	ns
	Leaves	Aphids	ns	H < U^º^	ns	H < S***	ns	S > U***
	Leaves	Whiteflies	ns	ns	ns	ns	ns	ns
Predators	Leaves	*Orius* spp.	H > U**	ns	H > S**	ns	ns	ns
	Flowers	*Orius* spp.	H < U***	ns	H > S^º^	ns	S < U***	ns
	Leaves	Phytoseiids	ns	ns	ns	ns	S > U*	ns
	Leaves	Thrips	ns	ns	ns	ns	ns	ns
	Flowers	Thrips	ns	H > U^º^	H > S**	H > S^º^	S < U*	ns
	Leaves	Cecidomyiids	ns	ns	ns	ns	ns	ns

(ns, not significant) P ≥ 0.1; (º) P < 0.1; (*), P < 0.05, (**) P < 0.05, (***) P < 0.01.

#### Abundance and population dynamics of natural enemies in melon fields

*Orius* spp. (Hemiptera: Anthocoridae) were the most abundant natural enemies observed on leaves. *Orius* spp. represented 74.7% of the predators, followed by phytoseiids (Acari: Phytoseiidae) (11.1%), predatory thrips (Thysanoptera: Thripidae and Aeolothripidae) (7%), gall midges (Diptera: Cecidomyiidae) (5.3%), predatory mirids (i.e., *Deraeocoris serenus* (Douglas & Scott)—Hemiptera: Miridae—) (1.2%) and lady beetles (Coleoptera: Coccinellidae) (0.5%). Chrysopid eggs were occasionally observed but not larvae. Parasitism rates of aphids were very low (0.2%). The absolute abundances for the main natural enemies in margins are summarised in the Supplementary Table S2.

**Figure 4:**
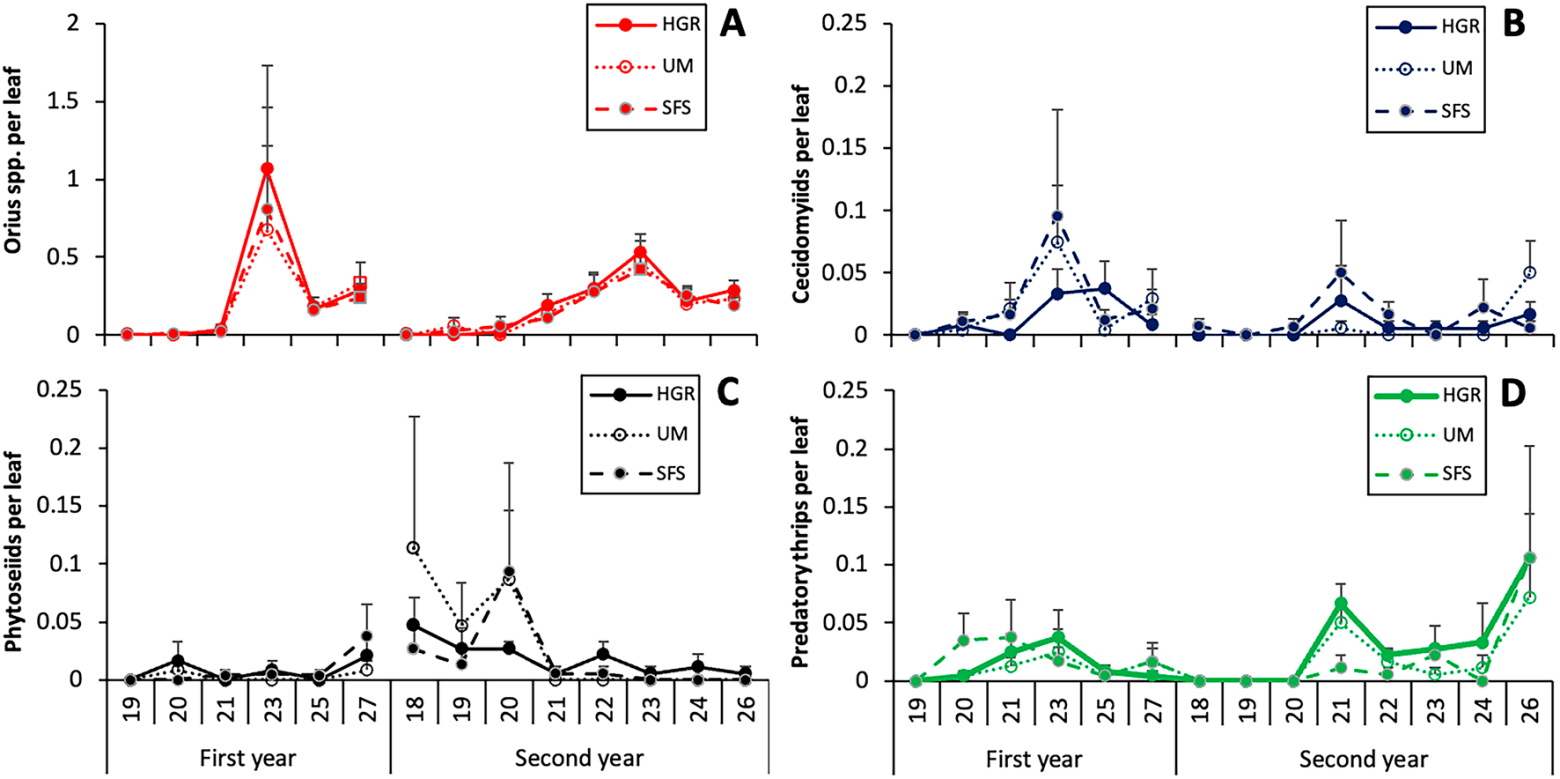
Abundance of the main natural enemies observed in the leaves of melon plants in fields with unmanaged floral strips (UM), managed floral strips (SFS) and hedgerows (HGR), expressed as the average number of individuals per leaf ± SE: *Orius* spp. (**A**), cecidomyiids (**B**), phytoseiids (**C**) and predatory thrips (**D**). Numbers above year’s labels indicate weeks.

In the sampling of leaves, the genus *Orius* was represented by the species *Orius laevigatus* (Fieber) and *Orius albidipennis* (Reuter) (Hemiptera: Anthocoridae), nymphs (90%) being much more abundant than adults (10%). Type of margin had a significant effect on the abundance of *Orius* spp. (χ^2^ = 15.2; df = 2; P < 0.001). In both years, the density of *Orius* spp. peaked on the 23rd week (Fig. 4A). In the sampling of flowers, two species of the *Orius* genus were observed: *O. albidipennis* (74.2%) and *O. laevigatus* (25.8%). In contrast to the leaf sampling, the percentage of adults (62.7%) in flowers was higher than that of nymphs (37.3%). Type of margin (χ^2^ = 39.9; df = 2; P < 0.001) and year (χ^2^ = 15.2; df = 1; P = 0.046) had a significant effect on the abundance of *Orius* spp. in flowers. In the first year, density peaks of 0.75 ± 0.18, 0.46 ± 0.17 and 0.40 ± 0.28 individuals per flower were observed in the melon fields with UM, HGR and SFS, respectively. In the second year, the density peaks were below 0.31 ± 0.05 individuals per flower.

Gall midges on leaves were represented by two species, *Feltiella acarisuga* (Vallot) (60%) and *Aphidoletes aphidimyza* (Rondani) (Diptera: Cecidomyiidae) (40%). Larvae were more abundant than adults (85% vs. 15%). The density of gall midges significantly differed between years (χ^2^ = 32.3; df = 1; P < 0.001), but no significant differences were observed among melon fields in relation to margin type (χ^2^ = 4.45; df = 2; P = 0.108) (Table 3). The abundance of gall midges usually peaked between weeks 21st and 23rd (Fig. 4B).

The phytoseiids found on melon leaves were *Phytoseiulus persimilis* (Athias-Henriot) and *Amblyseius* spp. (Mesostigmata: Phytoseiidae), the former (91%) being more abundant than the latter (9%). Significant differences in the abundance of phytoseiids were found between years (χ^2^ = 14.9; df = 1; P < 0.001), but not in relation to the type of margin (χ^2^ = 0.482; df = 2; P = 0.786), being the interaction between the two factors significant (χ^2^ = 6.56; df = 2; P = 0.038). In the first year, the abundance of phytoseiids peaked in the last week, while in the second year the peak took place during the firsts weeks (Fig. 4C).

Predatory thrips on leaves were represented by *S. longicornis* (63.4% of the predatory thrips adults) and *Aeolothrips* spp. (Thysanoptera: Aeolothripidae) (36.6%). The abundance of predatory thrips in melon fields significantly differed between years (χ^2^ = 6.56; df = 2; P = 0.038), but not in relation to the type of margin (χ^2^ = 5.38; df = 2; P = 0.068). The highest abundance of predatory thrips were reached between weeks 20th and 23rd in the first year and in the last week in the second (Fig. 4D)*. Aeolothrips* spp. were the only predatory thrips found in flowers and they were little abundant in comparison to phytophagous thrips (0.4% of the adult thrips). The abundance of predatory thrips in flowers was influenced by type of margin (χ^2^ = 6.21; df = 1; P = 0.013) and year (χ^2^ = 16.5; df = 2; P < 0.001). In the first year, density peaks of 0.13 ± 0.06, 0.12 ± 0.03 and 0.04 ± 0.03 individuals per flower were observed in the melon fields with HGR, UM and SFS, respectively. In the second year, density peaks were similar to those of the first year for the fields with HGR and SFS, but a little lower for fields with UM.

The abundance of predators in melon fields with different margin types differed significantly in just a few occasions and rarely there was consistency between the first and second year (Table 3). For instance, *Orius* spp. had a significantly higher abundance in melon fields with hedgerow than in those with sown floral strips and unmanaged margins in the first year, but in the second year no significant differences were found among treatments. Besides, discrepancies were found between the leaf and flower sampling (Table 3).

### Structure of the assemblage of pests and natural enemies in margins and melon fields with different vegetation margins

**Figure 5:**
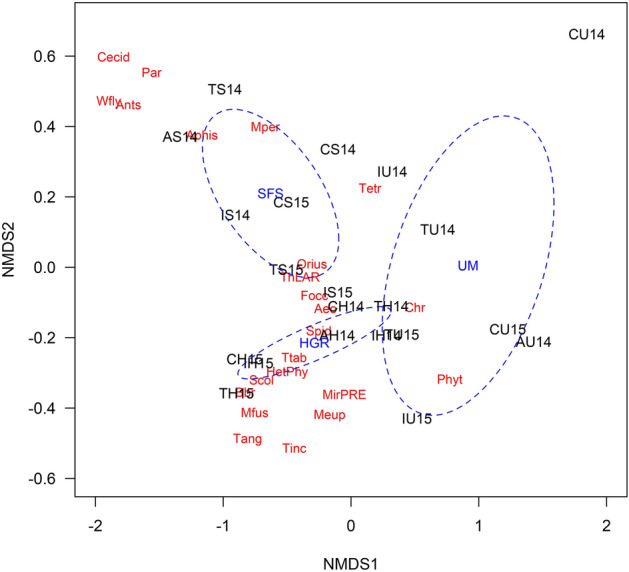
Non-metric multidimensional scaling (NMDS). Plot of the scores of the first two components for the melon fields in 2014 and 2015, and the main arthropods species (in red letters): Aeo (*Aeolothrips* spp.), Aphis (*Aphis* spp.), Ants (ants), Cecid (cecidomyiids), Chr (chrysopids eggs), Focc (*F. occidentalis*), HetPhy (Phytophagous Heteroptera), Meup (*M. euphorbiae*), Mfus (*Melanthrips fuscus*), MirPRE (predatory mirids), Mper (*Myzus persicae*), Orius (*Orius* spp.), Par (aphid mummies), Phyt (*Ph. persimilis*), Scol (*S. longicornis*), Spid (spiders), Tang (*Thrips angusticeps*), Tetr (*Tetranychus* spp.), ThLAR (thrips larvae), Tic (*T. inconsequens*), Ttab (*T. tabaci*) and Wfly (*B. tabaci*) Melons fields are shown in black, with the first letter indicating the locality—A (Arroyo), T (Torreblanca), I (IMIDA) and C (CIFEA)—, the second letter the margin type (U = UM, M = SFS and H = HGR) and the number the year. The blue ellipses represent the standard deviation (confidence interval 99%) for each margin type (UM = unmanaged floral strips, SFS = managed floral strips and HGR = hedgerows).

In the case of margins, NMDS shows an aggregation of the cases according to the type of margin (Fig. 5). The cases from managed margins (i.e., HGR and SFS) are mainly distributed on the negative side of the first axis and those from unmanaged margins on the positive side. Additionally, HGR cases are found on the negative side of the second axis, and SFS on the positive side (Fig. 5). Type of margin (R^2^ = 0.427, F = 9.99, df = 2, P < 0.001), year (R^2^ = 0.078, F = 3.64, df = 1, P = 0.016), and their interaction (R^2^ = 0.174, F = 4.08, df = 2, P = 0.002), had a significant effect on the abundance of arthropods. Sown floral strips were associated with several pest species, such as aphids, *Tetranychus* spp., thrips and whiteflies, and some natural enemies such as cecidomyiids and *Orius* spp. (Fig. 5). In contrast, hedgerows were associated with some phytophagous thrips that were little abundant or not found at all in melon crops such as *T. tabaci*, *T. angusticeps*, *M. fuscus*, *T. inconsequens,* and some predators such as predatory mirids, spiders and *Aeolothrips* spp. Unmanaged margins were associated to chrysopids and phytoseiids (Fig. 5). Significant differences were found in the pairwise comparisons between the three types of margins (Table 4).

**Table 4:** Summary of the pairwise comparison between types of margins.

Margin type 1	Margin type 2	df	SS	F-value	R^2^-value	P-value
HGR	SFS	1	0.348	3.16	0.209	0.049
HGR	UM	1	0.828	6.29	0.343	0.002
SFS	UM	1	1.273	10.3	0.463	0.002

df, degree of freedom; SS, sum of squares.

**Figure 6:**
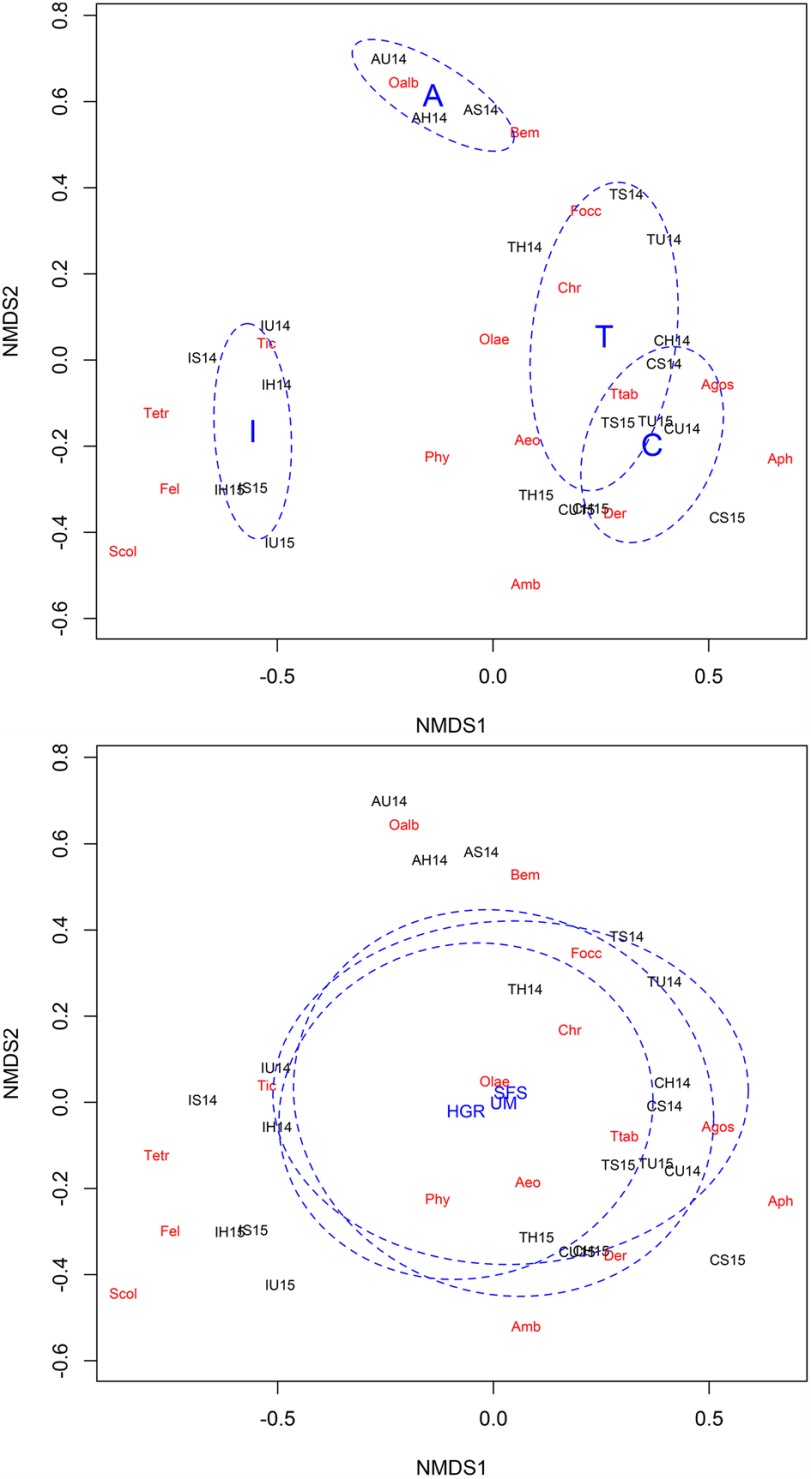
Non-metric multidimensional scaling (NMDS). Plot of the scores of the first two components for the melon fields in 2014 and 2015, and the main arthropods species (in red letters): Aeo (*Aeolothrips* spp.), Amb (*Amblyseius* spp.), Aph (*A. aphidimyza*), Bem (*Bemisia tabaci*), Chr (chrysopids eggs), Der (*D. serenus*), Fel (*F. acarisuga*), Focc (*F. occidentalis*), Oalb (*O. albidipennis*), Olae (*O. laevigatus*), Phy (*Ph. persimilis*), Scol (*S. longicornis*), Tic (*T. inconsequens*), Tetr (*Tetranychus* spp.) and Ttab (*T. tabaci*). Melons fields are written in black, with the first letter indicating the locality, the second letter the margin type (H = HGR, S = SFS and U = UM) and the number the year. Upper graph: the blue ellipses represent the standard deviation (confidence interval 99%) for each locality A (Arroyo), C (CIFEA), I (IMIDA) and T (Torreblanca). Lower graph: The blue ellipses represent the standard deviation (confidence interval 99%) for each margin type (HGR = hedgerows, SFS = managed floral strips and UM = unmanaged margins).

In the case of melon fields with different types of margins, NMDS showed an aggregation of the melon fields according to locality, but not to the type of margin, independently of the year (Fig. 6). The melon fields from the localities with more intensive agriculture (i.e., Torreblanca and CIFEA) grouped on the positive side of the first axis. PERMANOVA showed a significant effect of locality in the separation of the fields (R^2^ = 0.631, F = 6.48, df = 3, P < 0.001), while neither the effect of type of margin (R^2^ = 0.019, F = 0.297, df = 2, P = 0.951) nor the interaction (R^2^ = 0.204, F = 0.293, df = 6, P = 0.998) were found to be significant. The pairwise comparison showed significant differences between IMIDA and the rest of the localities, and also between Arroyo and CIFEA (Table 5). The highest dissimilarities were found between CIFEA-IMIDA (R = 0.747), and the lowest between CIFEA-Torreblanca (R = 0.190). In relation to the association between natural enemies and pests, specialist predators such as *F. acarisuga* and *S. longicornis* were associated to *Tetranychus* spp., the specialist *A. aphidimyza* to *A. gossypii*, and the generalist *O. laevigatus* to *F. occidentalis* (Fig. 6).

**Table 5:** Summary of the pairwise comparison between localities.

Loc. 1	Loc. 2	df	SS	F-value	R^2^-value	P-value
Arroyo	CIFEA	1	0.257	5.638	0.446	0.013
Arroyo	IMIDA	1	0.588	9.050	0.564	0.008
Arroyo	Torreblanca	1	0.270	2.392	0.255	0.095
CIFEA	IMIDA	1	1.547	29.469	0.747	0.002
CIFEA	Torreblanca	1	0.202	2.349	0.190	0.142
IMIDA	Torreblanca	1	1.355	13.596	0.576	0.003

Df, degree of freedom; SS, sum of squares.

### Yield in melon fields with different margin types

Yield significantly varied between years (χ^2^ = 85.8; df = 1; P < 0.001), but not among the melon fields with different types of margins (χ^2^ = 2.30; df = 2; P = 0.316). In the first year, yield was 8.9 ± 1.7, 8.3 ± 0.8 and 8.3 ± 1.1 kg per plant in the melon fields with hedgerows, managed and unmanaged floral strips, respectively. In the second year, yield was generally lower, being 5.9 ± 0.6, 5.3 ± 0.5 and 5.0 ± 0.3 kg per plant in the melon fields with managed floral strips, hedgerows and unmanaged floral strips, respectively.

## Discussion

Shrubby hedgerow and sown floral strips were found to harbour higher numbers of predators than unmanaged margins, but they also hosted higher numbers of several crop pests. Floral plantings may provide alternative prey/host, nectar, pollen, shelter, oviposition sites, etc. for natural enemies, but the same resources may also be used by pests for building up their populations^25,26,56–59^. Hedgerows and sown floral strips were found to differ in the assemblage and abundance of pests and enemies that they harboured. For example, some common vegetable pests (i.e. aphids, spider mites) were generally more abundant in herbaceous floral strips than in hedgerows. In addition, floral strips showed higher abundances of important pest thrips (e.g. *F. occidentalis*) than hedgerows. In relation to natural enemies, some groups such as predatory mirids, predatory thrips and spiders, were found to be more associated to shrubby hedgerows than to herbaceous margins. In contrast, the abundance of important predators, such as *Orius* spp., was high in both hedgerows and sown floral margins. It has been generally assumed that restored margins harbour a greater proportion of beneficial or neutral species than problematic ones^60^. However, the present research shows important differences in the assemblage and abundance of the arthropod species according to the composition of margins. Other authors have also pointed out the importance of choosing the right plant species for the design of margins in order to promote the most desirable species for the provision of effective ecosystem services^7,16,26,29,61,62^.

In the present research, in contrast with our working hypothesis, it was observed that melon fields with different margin types did not differ significantly in relation to their assemblages of arthropods. In just a few occasions margins were found to have a significant effect on the abundance of the different pests and natural enemy species in melon fields and rarely there was consistency between the first and the second growing season. The most consistent results were the higher abundance of spider mites in the melon fields with shrubby hedgerows than in melon fields with unmanaged margins. However, this is surprising because hedgerows were not found to differ from unmanaged margins in the abundance of spider mites. In relation to natural enemies, melon fields with hedgerows were found to have higher abundance of *Orius* spp. and predatory thrips in the first growing season, but not in the second. However, in the case of *Orius* spp. this was not conclusive because opposite results were obtained on leaf and flower sampling. In agreement with the results of the present research, some authors reported no correspondence between the abundance of predators in floral strips and adjacent melon fields^63,64^. Additionally, just a relatively low number of publications have conclusively reported increased natural enemy abundance and reduced pest populations in fields adjacent to floral plantings^28,31,65–69^.

The similarities on the assemblages of arthropods in melon fields from the same locality, independently of the margin type, could be due to the overriding effect of the landscape, among other variables. Landscapes with large pools of species have been hypothesised to override the effect of local practices on biodiversity and ecosystem services^4,70,71^. Many herbaceous and shrubby plant species common in Mediterranean agroecosystems host high populations of both pests and natural enemies that may serve as a source for the colonization of crops^7,72,73^. However the importance of migration from surrounding habitats may also depend on the characteristics of crops and the biology of the species^11^. For arthropod pests and predators that may entirely satisfy their requirements using crop food resources, immigration may be critical for the establishment of the initial population, but it may have a low contribution to population growth when the species starts reproducing and reaches high densities^1,2^. All the main phytophagous arthropods (i.e. aphids, thrips, spider mites and whiteflies) and natural enemies (namely *Orius* spp.) have high reproduction rates and undergo several generations per growing season in melons in southern Spain^40,74^. Therefore, we hypothesised that once these species have established and start reproducing in melon fields, in most of the cases, immigration could have a minor effect on the population growth. This is supported by the close association found between some specialist natural enemies such as *S. longicornis* and *F. acarisuga* and their prey, *Tetranychus* spp., as well as by the association between aphids and the specialist *A. aphidimyza* in melon fields. This is in agreement with what is expected from specialist predators as they closely follow the population dynamics of specific prey^75^. Generalist predators, such as *Orius* spp., were also found in close association with some of their preferred prey (i.e., *F. occidentalis*)^74^.

The evaluation of the impact of floral plantings on yield has remained largely unevaluated^36,38^. In the present research, yield was not found to differ significantly among melon fields with different types of margins. Several authors have also reported a lack of correlation between greening practices and yield increase in crops^28,29^.

In the present research it was found that restored margins hosted higher abundances of both pests and natural enemies than unmanaged margins, but no association was found between the assemblage of arthropods in margins and melon fields. Overall, melon fields with restored and unmanaged margins had similar pest and natural enemy abundances. We hypothesised that this could be due to the overriding effect of the landscape and/or to higher effect of the population growth rate of pests and natural enemies in the crops in relation to the immigration rates from external habitats after the initial colonization phase. The lack of differences in yield among melon fields with restored and unmanaged margins indicates similar levels of biocontrol and would not justify the investment of establishing green infrastructures if regarded from a strictly monetary point of view. However, it has to be taken into account that margins generally represent a small fraction of the landscape and it is likely that their effect would increase if floral plantings were more widespread and restoration practices were adopted at the community level. Previous works have shown that natural biocontrol is operating in melon fields, providing similar regulation of pest populations than chemical control and obvious revenues^40^. Therefore, more research is needed to assess which factors are driving natural biological pest control in agricultural landscape and to know the level of landscape degradation below which this ecosystem services could be compromised. If restoration practices are adopted, it is advisable to use shrubby plants because they usually have lower abundances of pest species and they harbour high populations of natural enemies than herbaceous plants. However, more research is needed to optimise the composition of floral plantings in order to favour natural enemies and to reduce pest populations.

### Supplementary Information

Supplementary Table S1.

Supplementary Figure S1.

Supplementary Tables.

## Data Availability

The analysed and generated datasets will be maintained at the IMIDA repository and will be available upon request.
