# Supplementary material for: How plant composition in margins influences the assemblage of pests and predators and its effect on biocontrol in melon fields
Source: Sci Rep. 2024 Jun 7;14:13094. doi: 10.1038/s41598-024-63985-x (PMC11161519; doi:10.1038/s41598-024-63985-x)
Supplement: Supplementary file 1 — Supplementary Table S1. [file 41598_2024_63985_MOESM1_ESM.docx]

|  |  | **Unmanaged margins** | | | | **Sown floral strips** | | | | **Hedgerows** | | | |
| --- | --- | --- | --- | --- | --- | --- | --- | --- | --- | --- | --- | --- | --- |
|  |  | **First year** | | **Second year** | | **First year** | | **Second year** | | **First year** | | **Second year** | |
| **Family** | **Plant species** | **%Bloss** | **%Green** | **%Bloss** | **%Green** | **%Bloss** | **%Green** | **%Bloss** | **%Green** | **%Bloss** | **%Green** | **%Bloss** | **%Green** |
| Apiaceae | *Conium sp.* |  |  | 0.5 | 0.6 | 0.0 | 0.0 | 0.0 | 0.0 |  |  |  |  |
|  | *Coriandrum sativum** |  |  | 0.1 | 0.3 | 0.2 | 2.6 | 0.2 | 2.6 |  |  |  |  |
|  | *Foeniculum vulgare* | 0.6 | 2.6 |  |  |  |  |  |  |  |  |  |  |
| Asteraceae | *Anacyclus clavatus* |  |  | 0.1 | 0.1 | 0.0 | 0.0 | 0.0 | 0.0 |  |  |  |  |
|  | *Calendula arvensis* | 0.7 | 1.2 | 0.5 | 1.1 | 0.0 | 0.1 | 0.0 | 0.0 |  |  |  |  |
|  | *Carduus sp.* |  |  | 0.0 | 0.1 | 0.0 | 0.1 | 0.0 | 0.1 |  |  |  |  |
|  | *Chrysanthemum coronarium** | 0.4 | 0.5 | 1.3 | 2.7 | 1.5 | 2.1 | 1.5 | 2.1 |  |  |  |  |
|  | *Matricaria chamomilla* |  |  |  |  | 0.0 | 0.0 | 0.0 | 0.0 |  |  |  |  |
|  | *Santolina chamaecyparisus*** |  |  |  |  |  |  |  |  | 0.7 | 5.7 | 4.2 | 11.0 |
|  | *Sonchus sp.* | 0.7 | 1.3 | 0.2 | 1.8 | 1.4 | 6.3 | 1.4 | 6.3 |  |  |  |  |
|  | *Taraxacum officinale* |  |  |  |  | 0.0 | 0.5 |  |  |  |  |  |  |
| Boraginaceae | *Borago officinalis** |  |  |  |  | 4.5 | 11.0 | 4.5 | 11.0 |  |  |  |  |
|  | *Echium vulgare** | 1.1 | 2.1 | 1.5 | 2.2 | 3.1 | 4.4 | 3.1 | 4.4 |  |  |  |  |
| Brassicaceae | *Brassica oleracea* |  |  |  |  | 0.3 | 0.6 | 0.3 | 0.6 |  |  |  |  |
|  | *Diplotaxis católica** |  |  |  |  | 0.0 | 0.0 | 0.0 | 0.0 |  |  |  |  |
|  | *Diplotaxis erucoides* | 0.3 | 0.5 | 1.7 | 2.5 | 0.0 | 0.0 | 0.0 | 0.0 |  |  |  |  |
|  | *Eruca vesicaria* | 0.0 | 0.1 | 0.0 | 1.0 |  |  |  |  |  |  |  |  |
|  | *Moricandia arvensis* | 0.3 | 1.5 | 0.1 | 0.2 | 0.0 | 0.1 |  |  |  |  |  |  |
|  | *Rapistrum rugosum* |  |  |  |  | 0.0 | 0.6 | 0.0 | 0.6 |  |  |  |  |
|  | *Sisymbrium irio* |  |  | 0.1 | 0.1 |  |  |  |  |  |  |  |  |
| Caryophyllaceae | *Silene vulgaris** |  |  |  |  | 3.9 | 16.5 | 3.9 | 16.5 |  |  |  |  |
| Chenopodioideae | *Beta sp.* | 0.0 | 0.8 |  |  | 0.0 | 2.5 | 0.0 | 2.5 |  |  |  |  |
|  | *Chenopodium album* |  |  | 0.0 | 0.2 | 0.6 | 2.2 | 0.6 | 2.2 |  |  |  |  |
| Cistaceae | *Cistus albidus* | 0.0 | 1.5 |  |  |  |  |  |  |  |  |  |  |
| Convolvulaceae | *Convolvulus spp.* | 0.7 | 1.8 | 0.3 | 0.3 | 0.3 | 0.7 |  |  |  |  |  |  |
| Cucurbitaceae | *Ecbacillum elaterium* | 0.5 | 3.8 | 1.8 | 4.8 | 0.1 | 0.5 |  |  |  |  |  |  |
| Cyperaceae | *Cyperus sp* | 0.0 | 0.0 |  |  |  |  |  |  |  |  |  |  |
| Euphorbiaceae | *Euphorbia spp.* | 0.0 | 0.0 | 0.0 | 0.2 |  |  |  |  |  |  |  |  |

**Supplementary Table S1.** %Bloss (Annual average percentage of blossoming) and %Green (Annual average percentage of green cover) in margins adjacent to melon fields.

|  |  | **Unmanaged margin** | | | | **Managed floral strips** | | | | **Hedgerow** | | | |
| --- | --- | --- | --- | --- | --- | --- | --- | --- | --- | --- | --- | --- | --- |
|  |  | **First year** | | **Second year** | | **First year** | | **Second year** | | **First year** | | **Second year** | |
| **Family** | **Plant species** | **%Bloss** | **%Green** | **%Bloss** | **%Green** | **%Bloss** | **%Green** | **%Bloss** | **%Green** | **%Bloss** | **%Green** | **%Bloss** | **%Green** |
| Fabaceae | *Bituminaria bituminosa* | 0.0 | 0.1 |  |  |  |  |  |  |  |  |  |  |
|  | *Dorycnium pentaphyllum*** |  |  |  |  |  |  |  |  | 2.2 | 7.3 | 10.9 | 17.9 |
|  | *Medicago sativa** |  |  |  |  | 0.5 | 0.7 | 0.5 | 0.7 |  |  |  |  |
|  | *Melilotus officinalis* |  |  | 0.1 | 0.4 | 0.5 | 1.1 | 0.5 | 1.1 |  |  |  |  |
|  | *Vicia sativa** |  |  |  |  | 0.2 | 4.9 | 0.2 | 4.9 |  |  |  |  |
| Fumariaceae | *Fumaria officinalis* | 0.0 | 0.1 | 0.0 | 0.2 | 0.0 | 0.0 | 0.0 | 0.0 |  |  |  |  |
| Geraniaceae | *Erodium sp* | 0.0 | 0.9 |  |  |  |  |  |  |  |  |  |  |
| Lamiaceae | *Ballota hirsuta*** |  |  |  |  |  |  |  |  | 0.0 | 0.3 | 0.0 | 0.0 |
|  | *Lavandula dentata*** |  |  |  |  |  |  |  |  | 3.7 | 7.8 | 6.3 | 12.4 |
|  | *Lavandula stoechas*** |  |  |  |  |  |  |  |  | 0.2 | 1.5 | 0.7 | 3.6 |
|  | *Phlomis purpurea*** |  |  |  |  |  |  |  |  | 0.0 | 1.7 | 0.1 | 3.9 |
|  | *Rosmarinus officinalis*** |  |  |  |  |  |  |  |  | 0.0 | 3.3 | 0.3 | 6.4 |
|  | *Salvia officinalis*** |  |  |  |  |  |  |  |  | 0.6 | 3.9 | 5.5 | 16.6 |
|  | *Salvia verbenaca** |  |  |  |  | 0.2 | 3.5 | 0.2 | 3.5 |  |  |  |  |
|  | *Thymus vulgaris*** |  |  |  |  |  |  |  |  | 0.2 | 2.1 | 0.5 | 1.2 |
| Malvaceae | *Lavatera sp.* | 0.0 | 0.1 | 0.0 | 0.5 | 0.0 | 0.3 |  |  |  |  |  |  |
|  | *Malva parviflora* | 0.0 | 0.1 | 0.0 | 0.2 | 0.1 | 3.6 | 0.1 | 3.6 |  |  |  |  |
| Plantaginaceae | *Plantago spp.* |  |  | 0.0 | 0.1 |  |  |  |  |  |  |  |  |
| Plumbaginaceae | *Limonium sp.* | 2.7 | 2.7 |  |  |  |  |  |  |  |  |  |  |
| Ranunculaceae | *Nigella damascena** |  |  |  |  | 0.0 | 0.0 | 0.0 | 0.0 |  |  |  |  |
| Resedaceae | *Reseda lutea* | 0.0 | 0.0 |  |  |  |  |  |  |  |  |  |  |
| Solanaceae | *Nicotiana glauca* | 0.3 | 1.5 |  |  |  |  |  |  |  |  |  |  |
|  | *Solanum nigrum* |  |  |  |  | 0.0 | 4.6 | 0.0 | 4.6 |  |  |  |  |
| Urticaceae | *Urtica urens* |  |  | 0.0 | 0.7 |  |  |  |  |  |  |  |  |
| Zygophyllaceae | *Fagonia cretica* | 2.5 | 4.5 |  |  |  |  |  |  |  |  |  |  |
|  | *Zygophyllum fagabo* | 0.2 | 0.3 |  |  |  |  |  |  |  |  |  |  |

**Supplementary Table S1, continuation.** %Bloss (Annual average percentage of blossoming) and %Green (Annual average percentage of green cover) in margins adjacent to melon fields. Sown herbaceous plant are marked with an asterisk and planted shrubby species are marked with two asterisks (**). Spontaneous plants are not marked.
