# Supplementary material for: How plant composition in margins influences the assemblage of pests and predators and its effect on biocontrol in melon fields
Source: Sci Rep. 2024 Jun 7;14:13094. doi: 10.1038/s41598-024-63985-x (PMC11161519; doi:10.1038/s41598-024-63985-x)
Supplement: Supplementary file 2 — Supplementary Figure S1. [file 41598_2024_63985_MOESM2_ESM.docx]

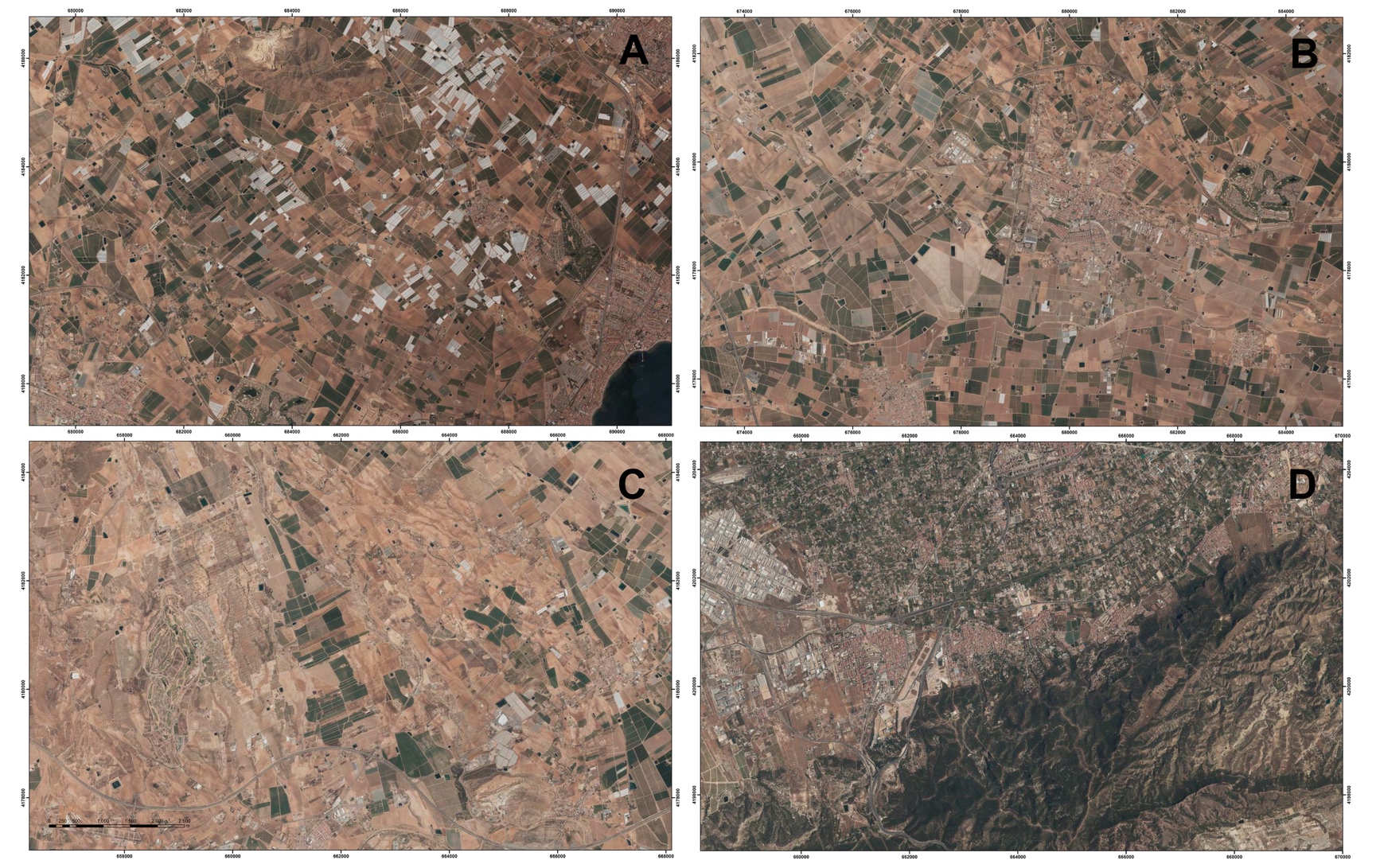


Supplementary Figure S1. Aerial photography of the landscape around the localities where the experiment was carried out. A, Dolores de Pacheco; B, Torre Pacheco ; C, Fuente Álamo and D, Alberca de las Torres.
