# Supplementary material for: How plant composition in margins influences the assemblage of pests and predators and its effect on biocontrol in melon fields
Source: Sci Rep. 2024 Jun 7;14:13094. doi: 10.1038/s41598-024-63985-x (PMC11161519; doi:10.1038/s41598-024-63985-x)
Supplement: Supplementary file 3 — Supplementary Tables. [file 41598_2024_63985_MOESM3_ESM.docx]

**Supplementary Table 2**. Total number individuals counted in three types of margins (hedgerows, HGR; sown floral strips, SFS; unmanaged margins, UM) in the visual and Berlese samplings.

|  |  |  | **HGR** | | **SFS** | | **UM** | |  |
| --- | --- | --- | --- | --- | --- | --- | --- | --- | --- |
| **Sampling** | **Type** | **Taxa** | **2014** | **2015** | **2014** | **2015** | **2014** | **2015** | **TOTAL** |
| **Berlese** | Natural enemies | *Aeolothrips* spp. | 75 | 28 | 308 | 117 | 1 | 78 | 607 |
|  |  | Ants | 0 | 38 | 0 | 37 | 0 | 17 | 92 |
|  |  | Araneae | 73 | 20 | 113 | 5 | 1 | 2 | 214 |
|  |  | Chrysopids | 1 | 0 | 1 | 0 | 0 | 0 | 2 |
|  |  | Coccinellids | 1 | 0 | 1 | 0 | 0 | 1 | 3 |
|  |  | *Orius* spp. | 340 | 418 | 175 | 177 | 1 | 80 | 1191 |
|  |  | Other Heteroptera Predators | 3 | 1 | 0 | 0 | 0 | 0 | 4 |
|  |  | Syrphids larva | 0 | 0 | 1 | 0 | 0 | 0 | 1 |
|  | Phytophages | Aphid non melon pests | 148 | 49 | 2917 | 358 | 29 | 474 | 2414 |
|  |  | *Aphis gossypii* | 42 | 54 | 14 | 5 | 1 | 1 | 117 |
|  |  | *Macrosyphum euphorbiae* | 0 | 0 | 0 | 10 | 0 | 43 | 53 |
|  |  | *Myzus persicae* | 6 | 8 | 226 | 61 | 55 | 55 | 411 |
|  |  | *Frankliniella occidentalis* | 2025 | 761 | 2568 | 1846 | 39 | 272 | 7511 |
|  |  | Thrips Non Pest (ad) | 349 | 250 | 5539 | 3806 | 26 | 1341 | 11311 |
|  |  | Thrips larvae | 1844 | 792 | 4070 | 5960 | 104 | 1788 | 14558 |
|  |  | *Tetranychus* spp. | 0 | 1 | 2 | 1 | 0 | 0 | 4 |
| Visual | Natural enemies | *Aeolothrips* spp. (ad) | 0 | 0 | 0 | 0 | 0 | 4 | 4 |
|  |  | *Scolothrips longicornis* (ad) | 0 | 2 | 0 | 59 | 8 | 68 | 137 |
|  |  | Ants | 3 | 1 | 4 | 25 | 134 | 15 | 182 |
|  |  | Araneae | 1 | 0 | 48 | 43 | 12 | 14 | 118 |
|  |  | Cecydomiids | 1 | 0 | 4 | 0 | 32 | 0 | 37 |
|  |  | Chrysopids (eggs) | 15 | 17 | 252 | 82 | 39 | 130 | 535 |
|  |  | *Orius* spp. | 7 | 1 | 24 | 25 | 46 | 11 | 114 |
|  |  | Other Heteroptera Predators | 2 | 0 | 20 | 9 | 8 | 3 | 42 |
|  |  | Phytoseiids | 0 | 9 | 0 | 8 | 0 | 35 | 52 |
|  | Phytophages | Aphid non melon pests | 49 | 25 | 124 | 58 | 1937 | 274 | 2467 |
|  |  | *Aphis* spp. | 7 | 0 | 12 | 0 | 249 | 4 | 272 |
|  |  | *Macrosiphum euphorbiae* | 0 | 103 | 0 | 204 | 0 | 224 | 531 |
|  |  | *Myzus persicae* | 59 | 62 | 4 | 47 | 354 | 198 | 724 |
|  |  | *Bemisia tabaci* | 1 | 6 | 4 | 8 | 213 | 25 | 257 |
|  |  | *Frankliniella occidentalis* | 4 | 0 | 36 | 3 | 46 | 19 | 108 |
|  |  | Thrips Non Pest (ad) | 175 | 31 | 151 | 40 | 265 | 32 | 694 |
|  |  | Other phytophagous thrips | 2 | 1 | 0 | 15 | 3 | 16 | 37 |
|  |  | Thrips larvae | 11 | 5 | 4 | 4 | 212 | 12 | 248 |
|  |  | Heteroptera phytophagous | 0 | 10 | 48 | 159 | 38 | 149 | 404 |
|  |  | *Tetranychus* spp. | 35 | 41 | 8 | 64 | 77 | 290 | 515 |

**Supplementary Table S3**. Total number individuals counted in melon fields in the visual and Berlese samplings.

|  |  |  | **HGR** | | **SFS** | | **UM** | |  |
| --- | --- | --- | --- | --- | --- | --- | --- | --- | --- |
| **Sampling** | **Type** | **Taxa** | **2014** | **2015** | **2014** | **2015** | **2014** | **2015** | **TOTAL** |
| **Berlese** | Natural enemies | *Aeolothrips* spp. (adults) | 37 | 19 | 19 | 8 | 36 | 11 | 130 |
|  |  | *Orius* spp. | 229 | 48 | 202 | 34 | 358 | 22 | 893 |
|  | Phytophages | *Aphis gossypii* | 48 | 7 | 60 | 31 | 80 | 20 | 246 |
|  |  | *Frankliniella occidentalis* (ad) | 5143 | 1344 | 5455 | 1320 | 6242 | 953 | 20457 |
|  |  | Thrips Non Pest (ad) | 704 | 138 | 343 | 119 | 436 | 104 | 1844 |
|  |  | Thrips larvae | 1493 | 789 | 2008 | 1029 | 2117 | 1088 | 8524 |
| **Visual** | Natural enemies | *Aeolothrips* spp. (ad) | 8 | 15 | 8 | 5 | 3 | 11 | 50 |
|  |  | *Scolothrips longicornis* (ad) | 11 | 31 | 21 | 21 | 12 | 17 | 113 |
|  |  | Cecydomiids | 22 | 11 | 38 | 19 | 32 | 10 | 132 |
|  |  | Chrysopids | 77 | 9 | 74 | 30 | 77 | 22 | 289 |
|  |  | *Orius* spp. | 412 | 279 | 326 | 239 | 308 | 247 | 1811 |
|  |  | Other Heteroptera Predators | 5 | 3 | 1 | 10 | 7 | 5 | 31 |
|  |  | Phytoseiids | 11 | 24 | 16 | 22 | 4 | 37 | 114 |
|  | Phytophages | *Aphis gossypii* | 1565 | 1085 | 1850 | 2546 | 1548 | 1253 | 9847 |
|  |  | *Myzus persicae* | 2 | 0 | 44 | 8 | 1 | 1 | 56 |
|  |  | *Bemisia tabaci* | 383 | 171 | 856 | 123 | 462 | 142 | 2137 |
|  |  | *Frankliniella occidentalis* (ad) | 1007 | 551 | 623 | 423 | 936 | 472 | 4012 |
|  |  | Thrips Non Pest (ad) | 161 | 103 | 100 | 83 | 150 | 100 | 697 |
|  |  | Thrips larvae | 5125 | 1940 | 2275 | 1588 | 4793 | 1698 | 17419 |
|  |  | *Tetranychus* spp. | 2961 | 6458 | 5376 | 4232 | 4609 | 2072 | 25708 |
